# Mechanotransduction channel Piezo is widely expressed in the spider, *Cupiennius salei*, mechanosensory neurons and central nervous system

**DOI:** 10.1038/s41598-021-87202-1

**Published:** 2021-04-12

**Authors:** Jessica A. G. Johnson, Hongxia Liu, Ulli Höger, Samantha M. Rogers, Kajanan Sivapalan, Andrew S. French, Päivi H. Torkkeli

**Affiliations:** 1 Department of Physiology and Biophysics, Dalhousie University, P.O. BOX 15000, Halifax, NS B3H 4R2 Canada; 2 Department of Biology, Mount Saint Vincent University, 166 Bedford Highway, Halifax, NS B3M 2J6 Canada

**Keywords:** Cell biology, Neuroscience, Cellular neuroscience, Sensory processing

## Abstract

Mechanosensory neurons use mechanotransduction (MET) ion channels to detect mechanical forces and displacements. Proteins that function as MET channels have appeared multiple times during evolution and occur in at least four different families: the DEG/ENaC and TRP channels, as well as the TMC and Piezo proteins. We found twelve putative members of MET channel families in two spider transcriptomes, but detected only one, the Piezo protein, by in situ hybridization in their mechanosensory neurons. In contrast, probes for orthologs of TRP, ENaC or TMC genes that code MET channels in other species did not produce any signals in these cells. An antibody against *C. salei* Piezo detected the protein in all parts of their mechanosensory cells and in many neurons of the CNS. Unspecific blockers of MET channels, Ruthenium Red and GsMTx4, had no effect on the mechanically activated currents of the mechanosensory VS-3 neurons, but the latter toxin reduced action potential firing when these cells were stimulated electrically. The Piezo protein is expressed throughout the spider nervous system including the mechanosensory neurons. It is possible that it contributes to mechanosensory transduction in spider mechanosensilla, but it must have other functions in peripheral and central neurons.

## Introduction

Mechanosensory cells detect force from signals such as touch, sound, vibration, and strain and convert these signals into ionic currents via mechanically activated ion channels. The molecular identities of mechanotransduction (MET) channels in many specialized mechanosensory cells remain unknown due to the lack of specific inhibitors and activators, difficulty of expressing them in heterologous systems, complex morphology of mechanosensory organs and the small number of channels in each cell^1^.

Members of four ion channel families have repeatedly been proposed as pore forming subunits of MET channels in eukaryotic cells: (1) The sodium selective amiloride sensitive degenerin/epithelial channel family (DEG/ENaC) comprising vertebrate ENaC and acid sensitive channels, *Drosophila* pickpocket (PPK) channels and *Caenorhabditis elegans* DEG channels^1–4^. (2) The Transient Receptor Potential (TRP) family including the *Drosophila* TRPN1 or NompC channel, which underlies mechanotransduction in bristle mechanosensilla, larval proprioceptors and touch sensitive cells^5–7^. NompC also contributes to hearing in the *Drosophila* Johnston’s organ^8^, but a heteromeric TRPV channel Nan/Iav is likely responsible for transduction in these cells^9–11^. Nan/Iav may also form the MET channels in locust auditory neurons^12^ and cockroach tactile spine neurons^13^. (3) Transmembrane Channel-Like proteins TMC1/2. Mutations in the human TMC1 lead to deafness, and deletion of mouse TMC1 removed MET currents in the inner ear hair cells^14,15^. When reconstituted into liposomes, the turtle TMC1 and budgerigar TMC2 form cation selective mechanosensitive channels^16^. The *C. elegans* TMC1 and TMC2 are involved in a variety of functions from egg laying to nociception and the *Drosophila* TMC protein functions in detecting food texture and in larval proprioception^17,18^. (4) Piezo proteins form cation selective mechanosensitive channels in expression systems^19,20^. Vertebrate Piezo1 is mainly expressed in non-neural cells and is involved in a variety of physiological functions from vascular homeostasis to cell regeneration, while Piezo2 is found in sensory neurons and specialized mechanosensory cells where its deletion leads to deficits in touch sensation and proprioception^19,21–25^. The *Drosophila* Piezo protein is expressed in many cells and required for larval mechanical nociception^26^.

Investigation of arthropod MET channels is often challenging due to the small size of model species and the difficulty of accessing their mechanosensory cells. However, mechanical and electrophysiological properties of the spider, *Cupiennius salei*, lyriform VS-3 slit sensillum have been extensively investigated^27–29^. This sensillum is in the anterior side of the leg patella. It consists of 7–8 cuticular slits that are shaped as a lyre, each innervated by a pair of mechanosensory neurons that are attached to a thin epithelium (hypodermis). Tips of the dendrites are attached to the slits; the cell bodies are within the patella and their axons extend to the leg nerve that contains hundreds of other sensory and efferent fibers^27,28,30^. Vibrations from potential prey, predators and mates, or the spider’s own movements distort the slits that hold the sensory dendrites^30^, leading to a Na^+^ driven MET current with transient and slow components^31,32^. This current was blocked by amiloride and gadolinium and enhanced by acidic pH^33,34^, suggesting that the MET channels may belong to ENaC family. However, these blockers are not uniquely selective to specific ion channels, and all putative MET channels are cation channels^1,4,35^.

Our aim was to identify members of putative MET channel families from *C. salei* transcriptomes, and to investigate their expression in the spider mechanosensory cells using in situ hybridization. Piezo was the only putative MET channel we found in these cells and it was also expressed in many central neurons. Therefore, we focussed our research on Piezo to begin to understand its functions in the arthropod peripheral and central nervous systems.

## Results

### Putative mechanotransduction channel sequences in *C. salei* hypodermis and CNS transcriptomes

The preparation of *C. salei* hypodermis and CNS transcriptomes has been previously described in detail^37^. For the hypodermis transcriptome, the thin epithelium (hypodermis) that is attached to the cuticle and leg muscle via tendons was collected from the coxa, femur, patella and tibia of a total of 56 legs^37^. The leg nerves, multiple different types of sensory neurons, efferent fibers, the surrounding glial cells, and epithelial cells are attached to the hypodermis^27,29,66^. The CNS transcriptome was made from two complete brain masses that are surrounded by muscle tissue and very likely also contained some muscle^37^. Therefore, both transcriptomes contained a variety of neural, glial, and muscle tissue as well as supporting and epithelial cells.

**Figure 1:**
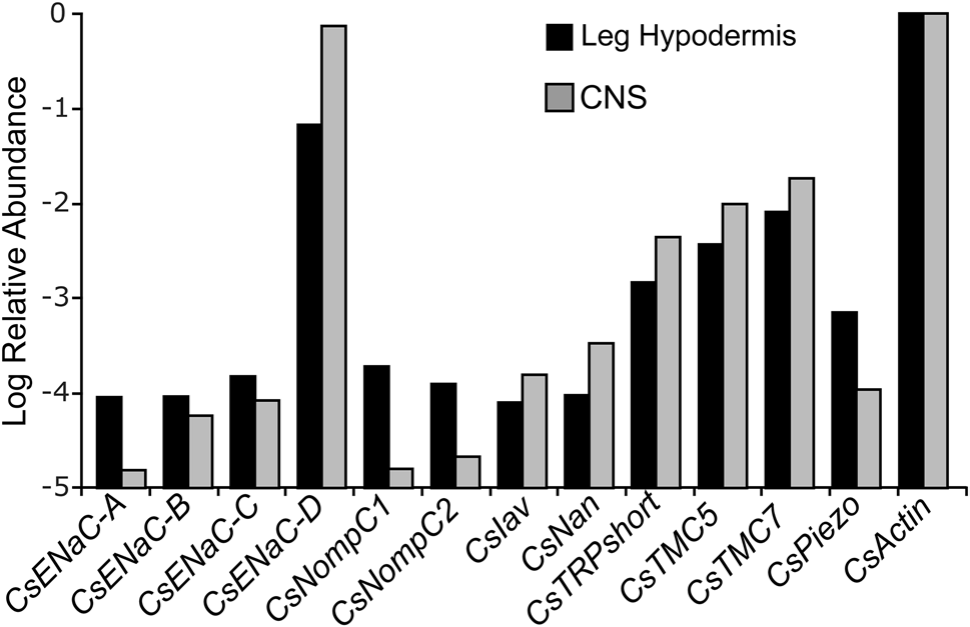
Relative abundances of putative mechanotransduction channel transcripts in *C. salei* leg hypodermis and CNS transcriptomes compared to the actin abundance. These data were obtained by counting total reads in the transcriptome libraries with at least 90 consecutive identical nucleotides to the reading frame of each gene, then normalizing by reading frame length. Note that the vertical scale is logarithmic.

The deduced proteins from twelve assembled mRNA sequences in *C. salei* leg hypodermis and CNS transcriptomes had conserved features of putative mechanotransduction channel families. We estimated the relative abundance of transcribed mRNA for each of these sequences by searching the transcriptome data for matches to the main open reading frame (mORF) as described previously in detail^36,37^. The total counts were normalized by mORF length and expressed as abundance relative to the putative actin coding sequence^36,37^. The relative abundances of each putative MET channel subunit in *C. salei* leg hypodermis and CNS transcriptomes are shown in Fig. 1. All twelve sequences were found in both tissues.

Four sequences (*CsENaC-A, B, C* and *D*) were homologous to the diverse amiloride sensitive DEG/ENaC superfamily that includes 31 *Drosophila* PPK subunits, 30 *C. elegans* DEG subunits, three mammalian ENaC and five ASIC subunits^2–4^. The similarities of deduced *C. salei* amino acid sequences were between 20 and 30% when compared to most of the *D. melanogaster* PPKs, *C. elegans* DEGs, and mouse ENaC and ASIC channels. The similarity of CsENaC-B was higher than other *C. salei* subunits when compared to any mouse ENaC or ASIC channels, or *C. elegans* and *Drosophila* subunits that have been shown to form mechanosensitive ion channels: *C. elegans* MEC4 31% and MEC10 29%, *Drosophila* ripped pocket (RKP) 28% and pickpocket 26 (PPK26) 26%. The *Drosophila* PPK1 was an exception and shared highest similarity with the CsENaC-D (26%). The *CsENaC-D* transcript was highly expressed in both the hypodermis and the CNS while the abundances of other ENaC subunits were significantly lower in both tissues. *CsENaC-A* was especially low in the CNS (Fig. 1).

InterPro scan and previous phylogenetic analysis^13^ indicated that two *C. salei* TRP sequences are orthologous to the insect TRPN or NompC channels and were named CsNompC1 and 2. We determined that these sequences share 90% similarity to each other, and 49% and 50% to the *Drosophila* NompC sequence. One sequence was orthologous with the insect Nan (CsNan). Another sequence was orthologous with insect Iav (CsIav). Our transcriptome analysis was not able to complete this gene, but the partial *Iav* gene has 421 amino acids. Both Iav and Nan are members of the TRPV family. The similarity of CsIav was 56% and the CsNan 57% to their *Drosophila* orthologs. Both *CsNompC* transcripts were more abundant in the hypodermis than in the CNS while the *CsIav* and *CsNan* were somewhat more highly expressed in the CNS than in the hypodermis (Fig. 1). The fifth TRP channel was homologous to the mammalian TRPC4, or short TRP (CsTRPshort). This transcript was the most abundant TRP sequence in the CNS and hypodermis. All *C. salei* TRP subunits, except the partial CsIav, are predicted to have an ankyrin repeat domain in their amino terminus that is believed to function as the MET channel gating spring^6^.

We found two transcripts that are predicted to encode proteins with the TMC motif and 9–10 transmembrane helices. One of these transcripts had the highest similarity (30%) to the mammalian subfamily that includes *TMC5* and *TMC6* genes^38^ and was named *CsTMC5*. The other transcript shares highest similarity with the mammalian *TMC7* and was named *CsTMC7*. We did not find any transcripts in the subfamily that includes mammalian *TMC1-3* genes and has one *Drosophila* and two *C. elegans* homologs^38^. Both *C. salei TMC* transcripts were relatively abundant in the CNS and hypodermis (Fig. 1).

We also found a transcript that is predicted to encode protein in the highly conserved Piezo family. The deduced CsPiezo protein has 33 transmembrane domains and a molecular weight of 295 kDa. A previous phylogenetic analysis placed CsPiezo in a clade with other Chelicerata, separate from insects and mammalian clades^13^. In pairwise analysis the CsPiezo similarity to both mouse Piezo1 and 2 proteins is 37% and to the *Drosophila* Piezo protein it is 38%.

### In situ hybridization probe for CsPiezo was the only putative MET channel probe that produced signal in *C. salei* mechanosensory cells

**Figure 2:**
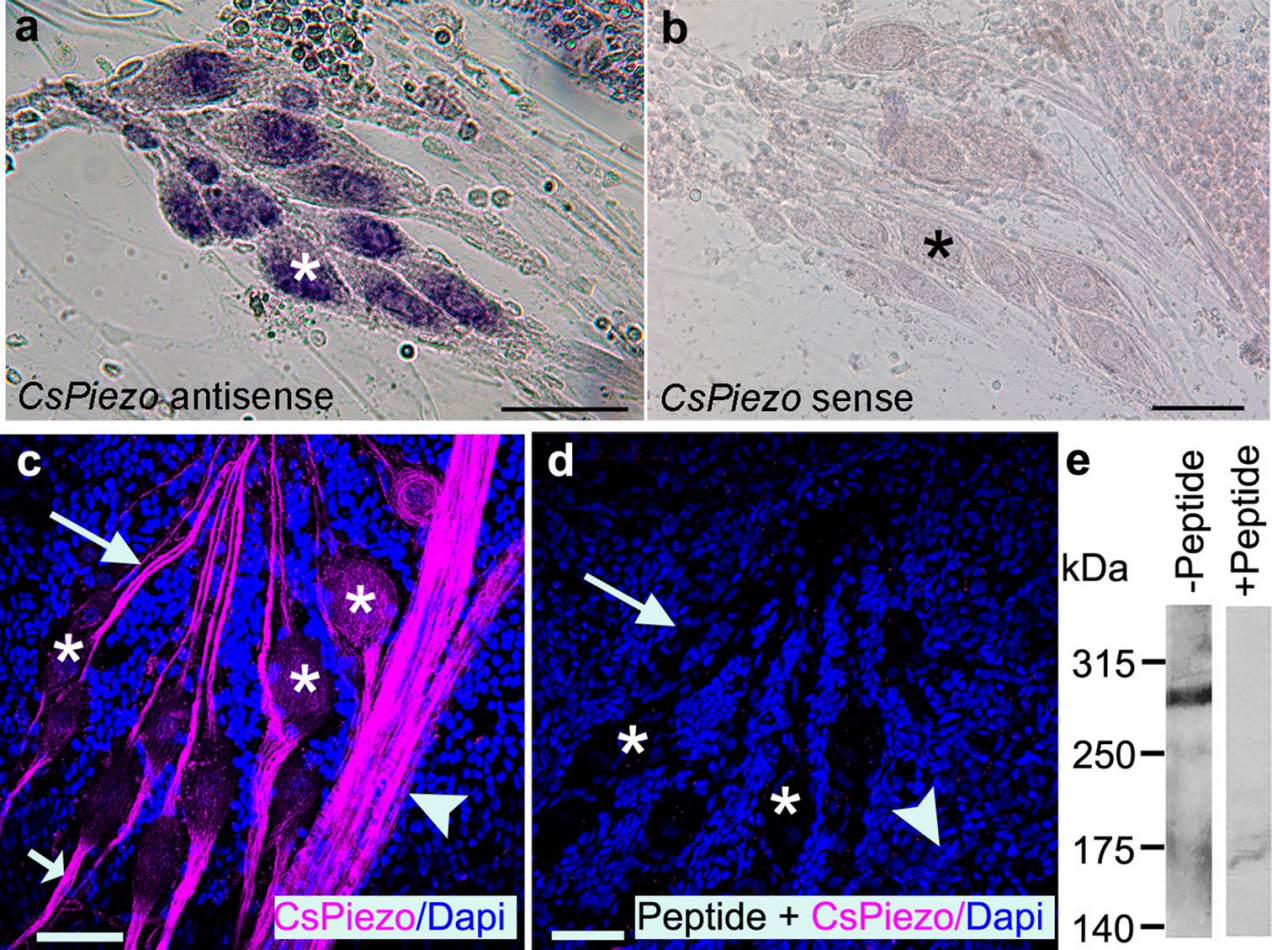
*CsPiezo* expression in *C. salei* VS-3 mechanosensory neurons. (**a**) In situ hybridization with a digoxigenin labeled antisense probe for *CsPiezo* mRNA produced strong signal in the mechanosensory VS-3 neuron somata (*one indicated with an asterisk*). (**b**) A sense (control) probe did not produce any labeling in VS-3 neurons or other cells of the patellar hypodermis. (**c**) A custom-made polyclonal antibody for CsPiezo protein (*magenta*) labeled VS-3 neuron dendrites (*large arrow*), axons (*small arrow*) and strong labeling was also present in the leg nerve (*arrowhead*). CsPiezo labeling in the central regions of the cell bodies (*asterisks*) was not as strong. Blue DAPI labeling shows the nuclei of many cells in the hypodermis. (**d**) The magenta labeling seen in the neurons in c was absent from control experiments where the CsPiezo antibody was incubated with an excess of the blocking peptide before the staining protocol. Arrow indicates the VS-3 dendrites, asterisks show some of the cell bodies and the arrowhead points to the leg nerve. DAPI staining is visible in many cells of the hypodermis. (**e**) The CsPiezo antibody produced a specific band at 295 kDa, the molecular weight of the CsPiezo protein. This band was not present in the control experiment where the primary antibody was preincubated with the blocking peptide (+ peptide) before the experiment. Scale bars in a-d are 50 µm.

To investigate if the putative MET channel transcripts are expressed in primary mechanosensory neurons, we used in situ hybridization and tested specific mRNA probes for each sequence (Supplementary Table 1) on a small piece of the patellar hypodermis. This tissue is similar to that used to create the transcriptome library, but all muscles and connections to the cuticle were removed. It contains multiple well characterized mechanosensory organs including the VS-3 slit sensillum, tactile spines, trichoid hair sensilla and joint receptors^27^. The sensory neurons are surrounded by glial cells and a complex array of efferent nerve fibers^39^. Of all the 12 antisense probes, only the *CsPiezo* produced a signal in all different types of mechanosensory neurons in spider patellar hypodermis. Figure 2a shows specific staining in the VS-3 neuron somata when the antisense *CsPiezo* probe was used while the surrounding glial and pigment cells have no staining. The sense (control) probe did not produce any signal in VS-3 neurons or other cells in the patellar hypodermis (Fig. 2b). All other eleven probes were tested in multiple preparations using different probe concentrations, hybridization temperatures and lengths of times for dye reaction. None of these antisense or sense probes produced specific staining in any of the mechanosensory neurons or other cells in the patellar hypodermis. It is possible that other parts of the legs have different types of sensory neurons that may express some of these transcripts and some of them are likely expressed in the muscle or tendons. Because of these results we focused on the *CsPiezo* sequence.

### The CsPiezo protein

**Figure 3:**
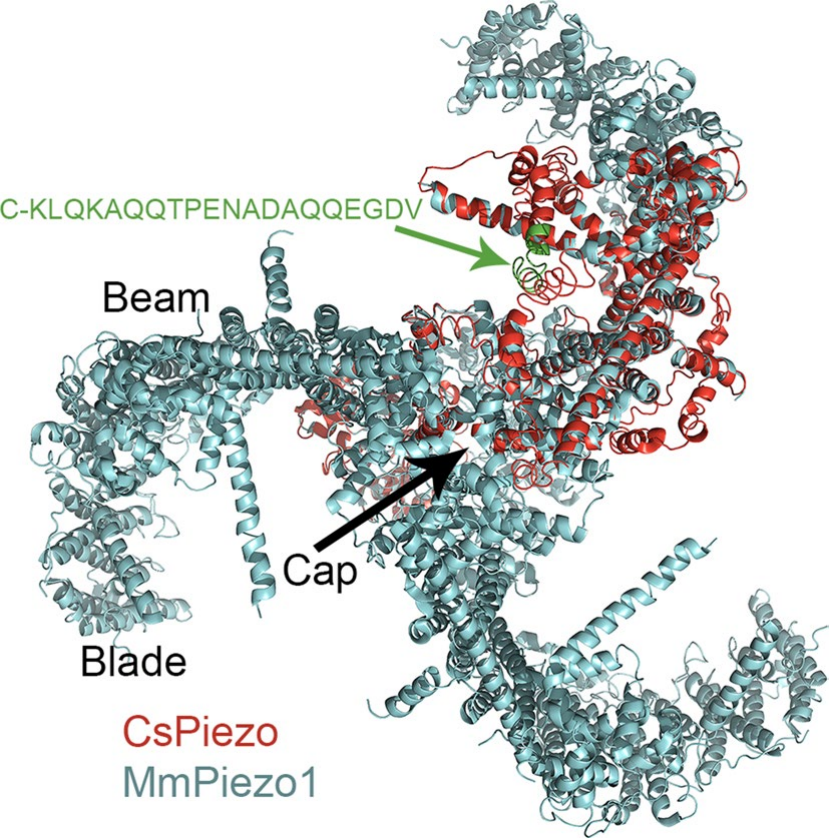
Homology model of the C-terminal half of *C. salei* Piezo protein. The model was created by the I-TASSER server and is shown in red as a cartoon representation aligned with one of the three subunits of the crystal structure of mouse Piezo1 (PDB 6B3R)^42^ shown in cyan. The structures are shown as viewed from the intracellular side. The MmPiezo1 is a trimeric, propeller-shaped structure that comprises the central cap, three peripheral blades and three long intracellular beams. The peptide sequence shown in green was used as an antigen to create an antibody for the CsPiezo protein.

We compared the structural similarity of the CsPiezo to templates available in the protein data bank (PDB) using the I-Tasser server^40^. The sequence has 2,571 amino acids and is too long to be submitted as a single file, therefore, we submitted it in two parts, the N-terminal residues 1–1300 and the C-terminal residues 1293–2571. Each sequence produced five models and those that were the best quality according to I-Tasser analysis, were selected for alignment. For the model based on N-terminal amino acids, the software used only one template, the mouse Piezo2 (PDB code 6KG7)^41^. For the model based on C-terminal residues, the main template chosen by the software was the mouse Piezo1 (6B3R)^42^ while mouse Piezo2 was less significant. We attempted to align the predicted CsPiezo models with the mouse Piezo templates using Pymol software (Schrödinger, LLC.). The N-terminal model did not align well with either template probably because part of this region in the mouse Piezo1/2 is still structurally unresolved^43^. The C-terminal part was a good fit with both mouse Piezo models and somewhat better match with the Piezo1. Mouse Piezo proteins consist of three identical subunits that form a three-bladed, propeller-like design, including a central ion-conducting pore at the C-terminal region, and three peripheral propeller blades^42,43^. Figure 3 shows the intracellular view of cartoon representation of all mouse Piezo1 subunits in cyan and the C-terminal half of CsPiezo model aligned to a mouse Piezo1 subunit in red. The amino acid sequence (residues 1493–1502) indicated in green in Fig. 3 was used to produce an antibody for the CsPiezo protein. Based on the homology model and prediction of the protein transmembrane helices by TMHMM (https://services.healthtech.dtu.dk/service.php?TMHMM-2.0,) this region is on the cytoplasmic side.

### CsPiezo protein is expressed in all parts of *C. salei* mechanosensory neurons but not in the peripheral efferent fibers

Specificity of the CsPiezo antibody was tested by Western blot using homogenate of the *C. salei* CNS. Figure 2e shows a single band at 295 kDa consistent with the predicted molecular weight of the CsPiezo protein. When a similar CNS sample was tested with CsPiezo antibody that was preincubated with the blocking peptide, no bands were detected, indicating that the effectiveness of the antibody was eliminated, and this antibody detected correct antigen in the CNS homogenate.

In immunocytochemistry, the CsPiezo antibody labeled all mechanosensory neurons of the patellar hypodermis. Figure 2c shows labeling in neurons of the VS-3 slit sensillum. Labeling was especially strong in the dendrites and axons, and some labeling was also visible in the cell somata. The leg nerves were also brightly stained. DAPI counterstain revealed many nuclei of non-neural cells of the hypodermis but these cells were not immunoreactive to CsPiezo. CsPiezo immunoreactivity was absent when the antibody was treated with the blocking peptide before it was used in the protocol (Fig. 2d).

**Figure 4:**
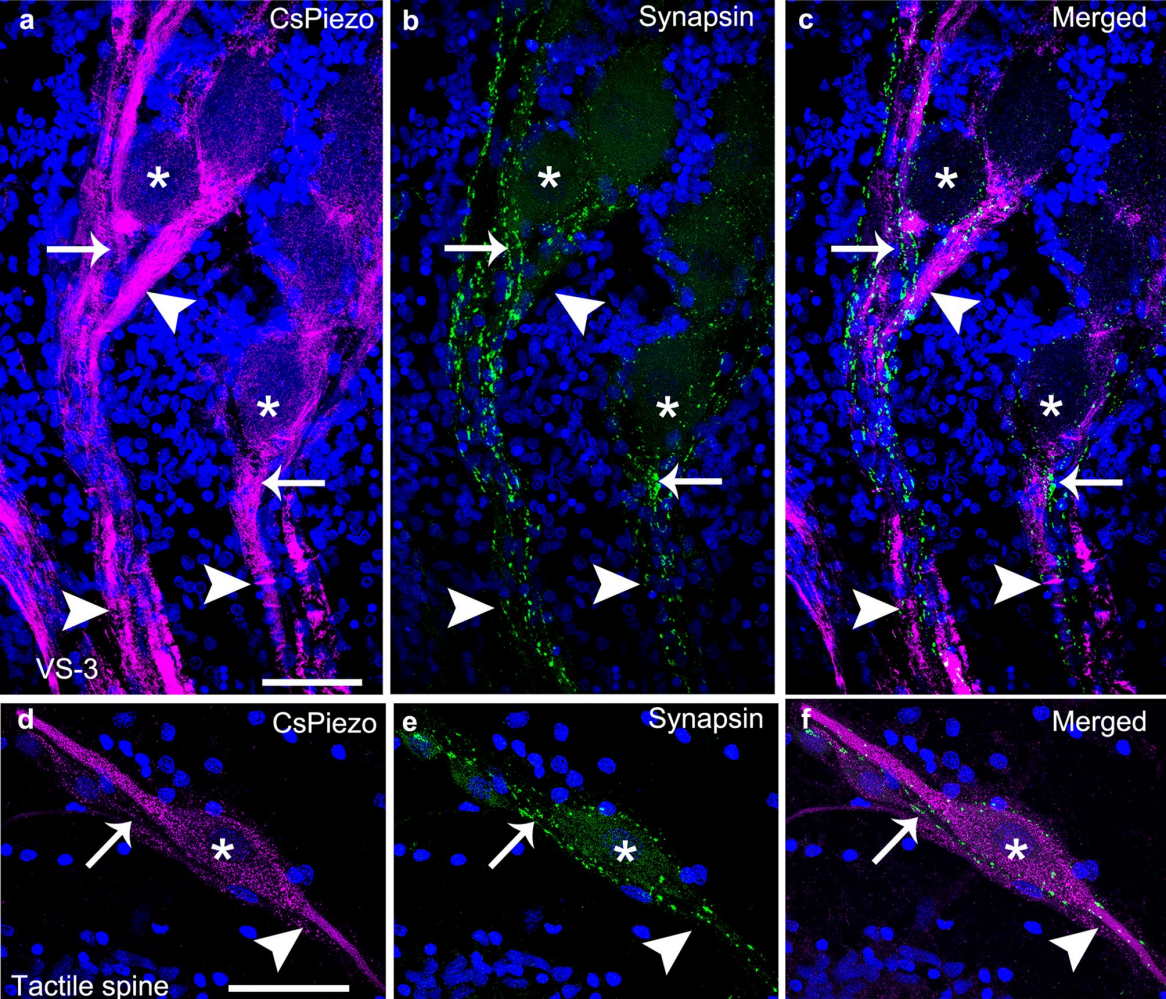
Double labeling with CsPiezo and synapsin antibodies. (**a–c**) The VS-3 neuron axons (*arrowheads*) were strongly labeled by the CsPiezo antibody (**a**, *magenta*) and the synapsin labeling (**b**, *green*) was in the fine efferent fibers that surround the axons and cell bodies (*some indicated by asterisks*). The merged image (**c**) shows that synapsin and CsPiezo were not co-localized (*arrows*), although the labeled structures were close to each other. (**d**,**e**) CsPiezo labeling (**d**) in the dendrites (*arrow*) and axons (*arrowhead*) of three neurons innervating a tactile spine in the spider patella. The three cell bodies (*the largest indicated by an asterisk*) were less strongly labeled. Synapsin labeling (**e**) in the efferent fibers was not co-localized with CsPiezo labeling, as seen in hte merged image (**f**) (*arrow*). Blue DAPI staining is in the nuclei of all cells of the hypodermis. Scale bars are 50 µm.

The spider mechanosensory neurons are surrounded by several thin efferent fibers^39^. To investigate whether these nerve fibers were also immunoreactive to CsPiezo, we performed double labeling experiments with the CsPiezo and a previously tested antibody against synapsin that labels synaptic vesicles in the efferent fibers^44^. Anti-synapsin labeled clusters were seen around all neurons of the hypodermis, including the VS-3 neurons (Fig. 4a–c) and the tactile hair neurons (Fig. 4d–f). This labeling is in proximity to the Piezo labeling, but there is no overlap of the magenta and green labeling (this would be white). If the Piezo expression were as extensive as it is in the sensory neurons, this would be seen as magenta lines joining the synaptic vesicles. Therefore, the two antibodies likely labeled different structures suggesting that the efferent fibers were not immunoreactive to CsPiezo.

### CsPiezo is widely expressed in nerve cells of *C. salei* CNS

**Figure 5:**
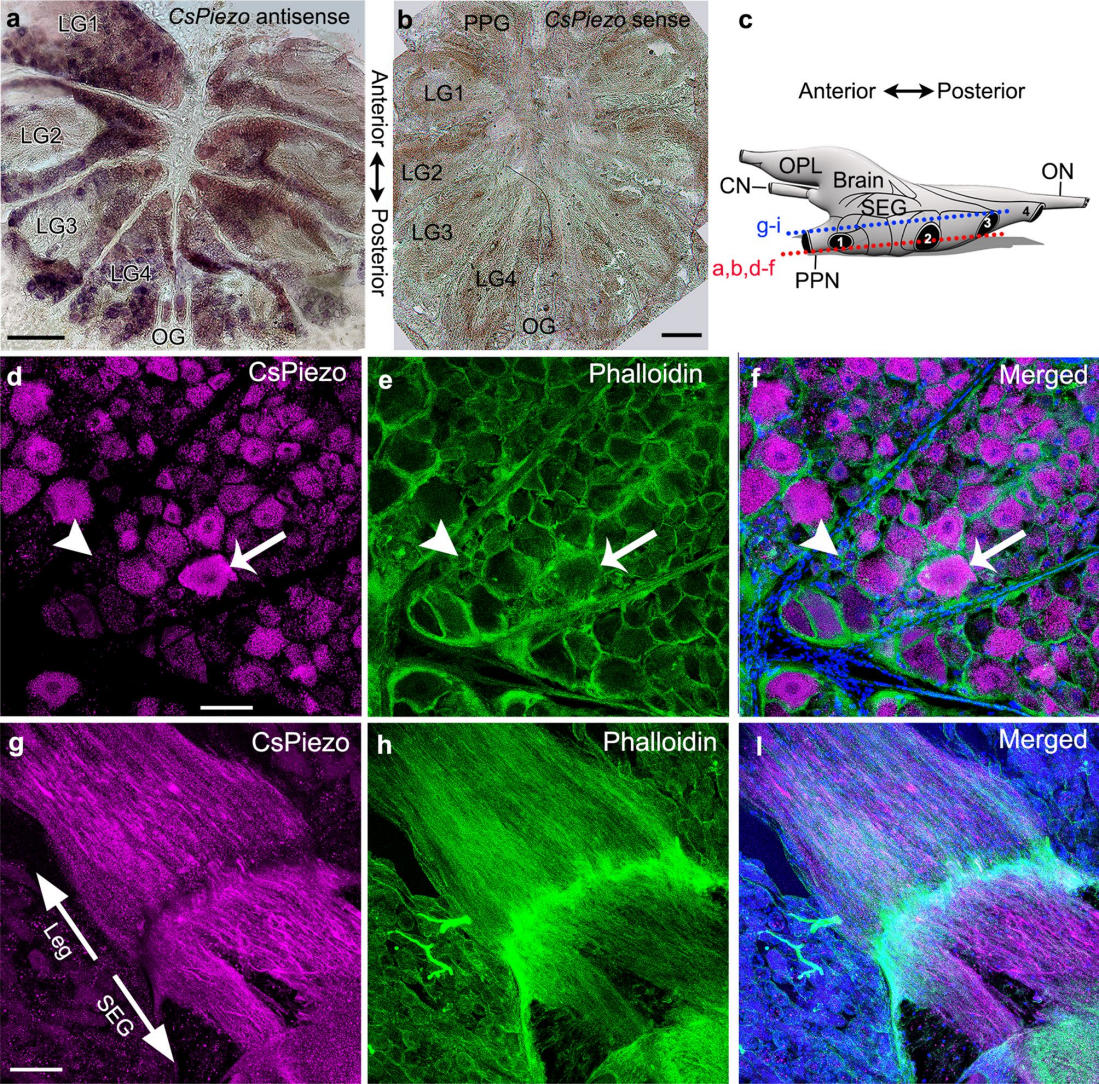
CsPiezo expression in the *C. salei* subesophageal ganglia (SEG). (**a**) An antisense probe for *CsPiezo* produced a strong signal in the cell bodies of many neurons located in the ventral region of the SEG. **b**. The sense (*control*) probe did not produce any signal. (**c**) Schematic drawing depicting a lateral view of the spider brain mass^45^. Approximate locations of the cross sections are indicated with red (a, b, d-f) and blue (g-i) dashed lines. (**d–f**) CsPiezo antibody (**d**, *magenta*) labeling in the somata of many neurons in the leg ganglia in the ventral SEG. Arrow points to one strongly labeled large soma. Phalloidin staining (**e**, *green*) in the actin filaments reveals many neurons that were not labeled with CsPiezo (*arrowhead*), or the labeling was weak. (**g–i**) The leg nerves are found in the dorsal region of SEG which is devoid of neuronal cell bodies. The leg nerves were strongly labeled by CsPiezo (**g**) and Phalloidin (**h**). Blue DAPI staining in merged images **f** and **i** in cells surrounding the leg nerves. Scale bars in a and b are 200 µm, in d–i 100 µm. LG = leg ganglia, OG = opisthosomal ganglia, ON = opisthosomal nerve, PPG = pedipalpal ganglia, OPL = optic lobes, CN = cheliceral nerve, PPN = pedipalpal nerve.

To find the extent of CsPiezo expression in the spider nervous system, we tested the *CsPiezo* probe and the CsPiezo antibody on horizontal sections of the *C. salei* CNS. The spider subesophageal nerve mass consists of several paired ganglia (four leg ganglia, pedipalpal and opisthosomal ganglia), the ventral region of each ganglion contains many cell bodies, varying in size from < 10 µm to more than 100 µm^45^. The antisense RNA probe for *CsPiezo* produced strong signal in many of these neurons while no signal was detected in any parts of the CNS when the control sense probe was used (Fig. 5a,b). Similarly, the CsPiezo antibody labeling was strong in many neurons in all subesophageal ganglia (Fig. 5d). The antibody labeling was not equally strong throughout the full thickness of the cells and the largest neurons were particularly weakly stained in some sections, while stronger staining was seen in sections above and below, suggesting that the plasma membrane was specifically labeled. Identities of most spider central neurons are not known, but most of the largest neurons are motor neurons^45^. Counterstaining with phalloidin for actin filaments and DAPI for nucleic acids revealed many small neurons that were not labeled by CsPiezo (Fig. 5e,f). Moreover, the nonneural cells that line each ganglion were not labeled by CsPiezo.

Nerves projecting to the periphery and other parts of the CNS are found in more dorsal regions of the spider subesophageal mass while the number of nerve cell bodies is very low in these areas^45^. The CsPiezo antibody labeling was very strong in the nerve branches, as can be seen in the leg nerves shown in Fig. 5g–i with phalloidin and DAPI counterstaining.

The spider supraesophageal nerve mass, or brain, contains the syncerebrum and cheliceral ganglia and has prominent optic lobes^45^ (Fig. 5c). We did not investigate these areas systematically but saw CsPiezo labeling in many of the neurons located in these dorsal areas of the CNS. Our results show clearly that expression of the Piezo protein is not limited to mechanosensory neurons but is also present in many central neurons. It is also evident that CsPiezo labeling is restricted to nerve cells.

### MET channel inhibitors Ruthenium Red and GsMTx4

**Figure 6:**
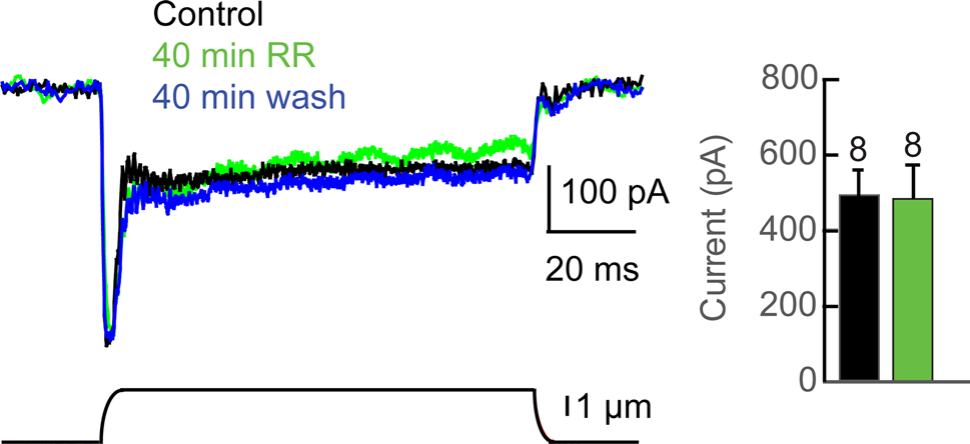
Ruthenium Red (RR) effect on mechanotransduction current. Step mechanical stimulus under voltage clamp induced inward current in a VS-3 neuron that was superfused in spider saline with 1 µM TTX (*black trace*). When 10 µM Ruthenium Red was added to the bath solution, the current remained unchanged (*green trace*) and similar current was also recorded after 40 min wash (*blue trace*). Bar graph shows the peak currents from 8 similar experiments under control conditions and 40 min after Ruthenium Red was added to the bath solution. There was no statistically significant difference between the MET currents (one tailed paired t-test *p* = 0.42735).

**Figure 7:**
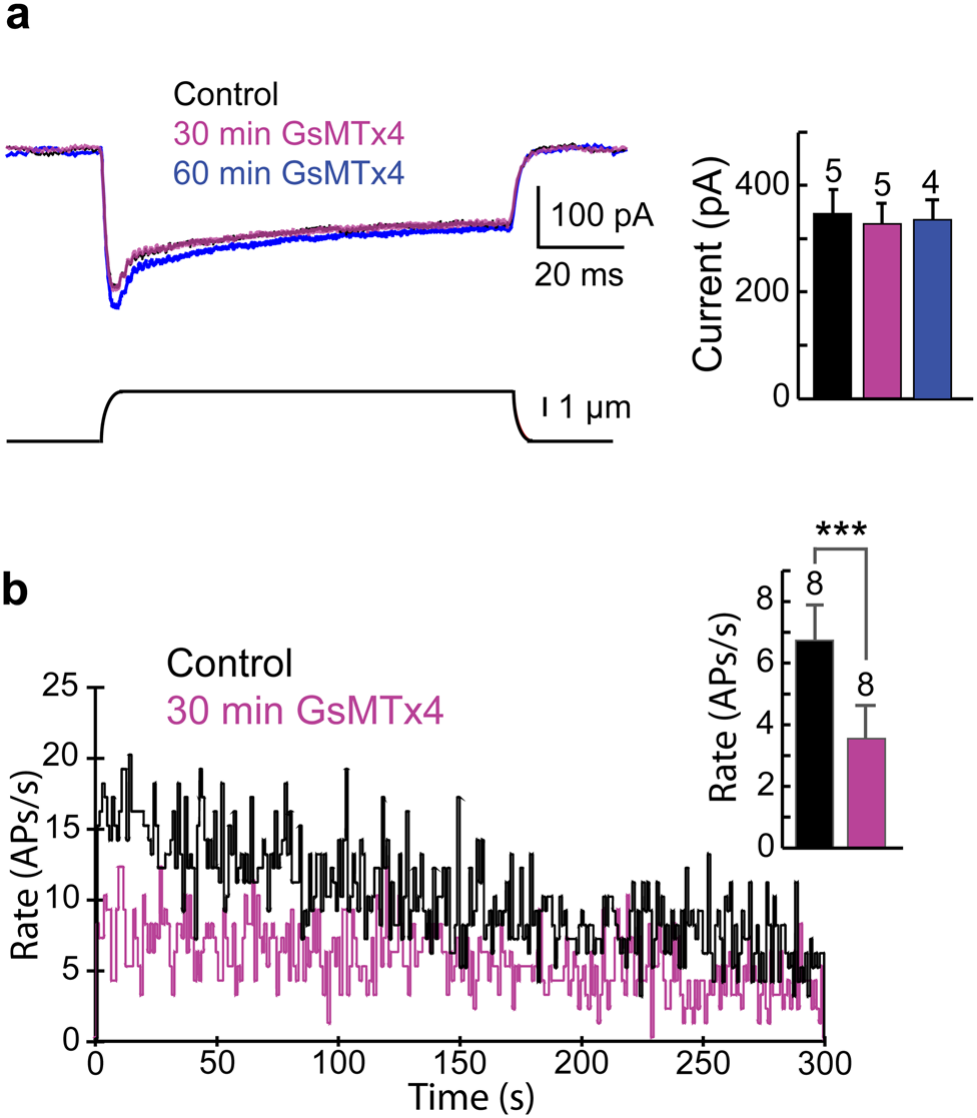
GsMTx4 effects on VS-3 neuron mechanotransduction current and action potential firing rate. (**a**) VS-3 neuron was superfused in spider saline with 1 µM TTX. Mechanical step stimulation under voltage clamp produced an inward current (*black trace*). Similar current was observed 30 min after 5 µM GsMTx4 was added to the superfusion solution (*purple trace*). In this experiment the current was slightly larger than control after 60 min superfusion in 5 µM GsMTx4. Bar graph shows the peak current amplitudes in five similar experiments. There were no statistically significant differences in the MET currents (one-tailed paired t-test *p* = 0. 0.368525). (**b**) Black trace is the action potential (AP) firing rate when the VS-3 neurons were superfused in normal spider saline. The purple trace is the AP firing rate 30 min after 5 µM GsMTx4 was added to the bath solution. APs were elicited by electrical pseudorandom white noise stimulation and counted to give AP firing rates. Bar graph shows the AP firing rates in 8 neurons under control condition and 30 min after GsMTx4 was added. The average AP firing rate was significantly lower when GsMTx4 was in the bath solution (paired one-tailed t-test *p* = 0.0004125).

Some Piezo channels are inhibited by unspecific blockers of mechanically activated ion channels, Ruthenium Red and/or the tarantula toxin GsMTx4^46^. We tested the effects of both blockers on *C. salei* VS-3 neuron mechanotransduction currents. Mechanical step stimulation of VS-3 neurons under voltage clamp produces a transient inward current followed by a sustained phase (Fig. 6). The VS-3 neuron MET current was unchanged in eight different preparations that were incubated in 10 µM Ruthenium Red for up to 40 min. Similarly, addition of 5 µM GsMTx4 to the bath solution did not reduce the MET current amplitude. In the example shown in Fig. 7a, the MET current was unchanged after 30 min in GsMTx4 and slightly larger than the control after one hour. Similar results were obtained in five different experiments. Taken together, these results suggest that either the *C. salei* Piezo channels are not sensitive to GsMTx4 and Ruthenium Red or other types of MET channels generate the MET current in the VS-3 neurons.

Since our immunocytochemical experiments indicate that the Piezo protein is located in all regions of mechanosensory neurons, we also tested whether GsMTx4 had any effects on the electrical properties of VS-3 neurons. We did not find any changes in membrane potential, membrane resistance or threshold for firing action potentials when the neurons were stimulated by step current stimuli. However, when the neurons were stimulated with pseudorandom white noise current for 5 min, the rate of firing action potentials decreased significantly in eight different experiments after GsMTx4 was added to the superfusion (Fig. 7b). White noise stimulation was used because it contains a wide frequency bandwidth similar to the signals that these neurons are expected to receive in their natural environment^47^. These results suggest that GsMTx4 blocked a current that allows VS-3 neurons to fire action potentials at a high rate.

## Discussion

In situ hybridization did not produce signals in any cells of the patellar hypodermis with probes for three ion channel families (DEG/ENaC, TRP and TMC). Thus, these subunits are unlikely to form MET channels in the mechanosensory cells in this tissue. The tissue for hypodermis transcriptome was collected from the coxa, femur, patella and tibia, that contain other sensory neurons and likely also some muscle tissue^37^. So, it is not surprising that the same mRNA that was found in the transcriptome was not detected in the specific cells on a small piece of patellar hypodermis that was used for in situ hybridization. CsPiezo was the only probe that produced clear signals in mechanosensory cells, but the antibody against CsPiezo labeled all regions of these cells and many neurons of the CNS indicating that the protein is probably not solely involved in sensory mechanotransduction.

The spider transcriptomes had only four DEG/ENaC subunits compared to *Drosophila* and *C. elegans* genomes that have 31 and 30 subunits, respectively^3^. It is possible that there are additional low abundance transcripts that we did not detect, and the spider opisthosoma could express many more DEG/ENaC genes. To our knowledge, the DEG/ENaC family has not been systematically investigated in any arachnid species and it is not known if they have as many homologous genes as insects and nematodes. Phylogenetic analysis has shown that invertebrate DEG/ENaC sequences are not orthologous to mammalian ENaCs or ASICs but they are in different subfamilies^4^. The DEG/ENaC/ASIC superfamily is a diverse group of proteins. Several form Na^+^ selective amiloride sensitive trimeric ion channels, and some are activated by acidic pH and/or mechanical stimulus^3,4^. Since the *C. salei* VS-3 neuron MET current is highly selective for Na^+^, inhibited by amiloride, and the channel open probability increased at acidic pH^31,33,34^, the DEG/ENaC family is a candidate for their MET channels. However, there are many other channels that allow Na^+^ influx and are sensitive to acid, and although amiloride is a potent blocker of DEG/ENaC channels, it also inhibits other ion channels and transporters^4,56,58^. Moreover, the effective amiloride concentration in *C. salei* VS-3 neuron experiments was 1 mM^33^ which is significantly higher than is needed to block mammalian ENaC and ASIC channels^4^ or *C. elegans* MEC4 channels^48^.

Different combinations of the TRP subunits NompC, Nan and Iav contribute to mechanotransduction in several insect sensory organs^5,7,9,11–13^. We found orthologs to all these channels in the spider transcriptome, but in situ hybridization suggests that they are not expressed in the patellar mechanosensory cells and are unlikely to form their MET channels. The sensory dendrites of spider slit sensilla project into a receptor lymph space, similarly to insect cuticular mechanoreceptors. In insects, this space has high K^+^ concentration that creates a transepithelial potential and the MET current is likely driven by K^+^^49^. The spider slit sensilla receptor lymph space has no transepithelial potential and its K^+^ and Na^+^ concentrations are similar to hemolymph while the Ca^2+^ concentration is three times lower in the lymph space and the MET current is driven by Na^+^^31,33,50^. These differences may have led to the use of different types of MET channels in insects and spiders.

The two TMC sequences that we found in *C. salei* transcriptomes are predicted to code proteins that are most similar to the mammalian TMC5 and TMC7, which are both in TMC subfamilies that are expressed at relatively high levels in multiple tissues^38^. They were also abundant in *C. salei* transcriptomes. We did not find any TMC transcripts in the subfamily that includes TMC1-3 that are expressed at low levels in mouse and include the two genes that are essential for their auditory transduction^18,38^. The only known *Drosophila* TMC protein is orthologous to this group and contributes to larval proprioception^17^, while *C elegans* has two orthologous proteins with a variety of functions^18^. It is possible that other parts of spider body have additional TMC genes or there may be very low abundance genes in the hypodermis and CNS. Interestingly, *Drosophila* or *C. elegans* do not have any known genes in the TMC4-8 groups^18,38^ but we have found two such transcripts in cockroach (*Periplaneta americana*) retina and antennae plus one in the locust *Schistocerca gregaria* Müller’s organ (unpublished work).

Transduction in arthropod cuticular mechanosensilla is believed to involve opening of a small number of MET channels located at the tips of the sensory dendrites^49^. However, CsPiezo antibody labeled the full length of the dendrites, axons and somata of all patellar mechanosensory cells, and many central neurons, indicating that although it may form MET channels in these cells, it must have other functions. Piezo proteins are broadly expressed in multiple cells and tissues in all animals that have been studied and they serve many functions from detecting shear stress in endothelial cells to cellular migration. Mammalian Piezo1 is mostly expressed in nonneural cells such as blood vessels, endothelial cells and internal organs^20,51,52^ while Piezo2 is more common in neurons but has also been found in nonneural cells^22,53,54^. *Drosophila* Piezo protein is expressed in all parts of larval and adult sensory neurons as well as nonneural tissues^26^. However, its role in mechanotransduction is limited to detecting noxious mechanical stimuli while transduction of touch and auditory signals are mediated by other proteins^5,26^. Moreover, the larval multidendritic cells that use Piezo to respond to noxious mechanical stimuli have another, parallel sensory pathway that uses the ENaC channel PPK^26^. Mouse Piezo2 is localized in the sensory endings of proprioceptors and various touch sensitive neurons in the skin and the mechanosensitivity of these cells depend partially on Piezo2^22,25^ but other unknown molecules are likely involved. Piezo2 is also expressed in the somata of these sensory cells in dorsal root ganglia and when grown in culture, they respond to mechanical stimuli^22,25,46^. However, their normal physiological role is unknown.

Unfortunately, the pharmacology of Piezo and other MET channels is poorly understood. The *C. salei* VS-3 neuron MET current is highly selective for Na^+^ over K^+^, which is different to the functionally expressed mammalian and *Drosophila* Piezo channels that are nonselective cation channels^19,46^. The VS-3 neuron MET current is blocked by relatively high concentrations of gadolinium and amiloride^32,33^. Neither of these blockers are specific; gadolinium inhibits Piezo1/2 channels^46^ but also many other types of channels that are gated by voltage or mechanical stimuli^33,35,55^. Amiloride is an effective inhibitor of DEG/ENaC channels but it also inhibits TRP channels and many transporters^4,56,58^. Similarly, Ruthenium Red is not particularly selective to Piezo proteins or MET channels, but it blocks many different types of ion channels^57,58^. The fact that it did not inhibit the MET current in VS-3 cells does not rule out Piezo as their MET channel. Although it inhibits expressed mammalian Piezo1/2, it has no effect on the *Drosophila* Piezo^19,46^. This may be explained by the replacement of residues E2495 and E2496 that are critical for mouse Piezo Ruthenium Red sensitivity, with E2466 and D2467 in *Drosophila* Piezo^59^ and D2502 and D2503 in CsPiezo. GsMTx4 blocks mechanosensitive cation channels including Piezos^60^. Although voltage activated K^+^ and Na^+^ channels are also sensitive to the L-form of GsMTx4 at relatively high concentrations^61^, lower concentrations of its D-form, which was used here, are unlikely to have significant effects on voltage gated channels^62^. GsMTx4 had no effect on the VS-3 neuron MET current, but it reduced the firing rate when they were stimulated electrically. If this effect was caused by the specific block of Piezo channels, it indicates that they can normally be activated by changes in voltage without mechanical stimulus. Piezo channels are voltage sensitive and although the biophysical properties of arachnid Piezo channels have not been investigated, insect Piezos are more like classical voltage gated channels than mammalian Piezos^63^. Another possible explanation is the mechanism of GsMTx4 inhibition; it partitions to the bilayer and inhibits by changing local membrane properties near the channel^60^. This could modify the activities of ion channels that generate action potentials.

In conclusion, our findings indicate that several candidate proteins are unlikely to form MET channels in *C. salei* mechanosensory cells, although other low abundance members of some of the same channel families may be present and may contribute to mechanotransduction. Our findings of Piezo protein show that it is specifically expressed in neurons throughout the spider central and peripheral nervous systems, and it must have important functions in these tissues. Due to its similarity to other Piezo channels, it is likely a MET channel and it is possible that it contributes to mechanotransduction in primary mechanosensory neurons. However. understanding its exact roles requires its electrophysiological characterization and the development of more specific inhibitors or silencing the Piezo gene using RNA interference or other molecular tools.

## Methods

### Experimental preparations

A colony of the Central American wandering spiders, *Cupiennius salei* (Keys.) was maintained at 22 ± 2 °C under a 13/11 h light/dark cycle. Adult spiders were used for experiments and the experimental protocols were approved by the Dalhousie University Committee on Laboratory Animals. For patellar preparations, legs were autotomized and placed on a Sylgard-coated dish. We dissected the anterior side of the patella and removed the muscle tissue to provide access to sensory neurons. For dissection of the CNS, spiders were anesthetised with CO_2_ and for Western blot analysis, the CNS was quickly dissected and placed in cold spider saline^33^. For in situ hybridization and immunocytochemistry, the tarsal leg segments were removed, and the anesthetized spider was perfused with 4% paraformaldehyde that was injected directly into the heart. The sternum plate was then removed, the CNS extracted and kept in 4% paraformaldehyde overnight.

### Identification and analysis of putative mechanotransduction channels from *C. salei* transcriptome

Details of the *C. salei* transcriptome preparation, sequencing and assembly have been described previously^37^. Twelve putative MET channel sequences were identified using the transcriptome walking algorithm^36^. Data analysis and programming were done using the C ++ language with Microsoft Visual Studio. The sequences were analyzed by the InterProScan 5 server (http://www.ebi.ac.uk/Tools/pfa/iprscan5/), which scans the sequence against the protein signatures of several databases. Pairwise identity matrices were created using a Sequence demarcation tool with the MAFFT aligned sequences^64^ and clustering the sequences with Neighbor joining tree^65^.

### Prediction of *C. salei* Piezo protein structure

We used the I-Tasser server^40^ to generate 3D-structure predictions of the *C. salei* Piezo protein. The sequence was uploaded in FASTA format in two parts (residues 1–1300 and 1293–2537) and both were run without restraints allowing the software to select the templates. We used the PyMOL Molecular Graphics System, Version 2.1.0. (Schrödinger, LLC.) software to generate images of the homology models.

### RNA Probe construction and in situ hybridization

Custom designed primers (Supplementary Table 1) were purchased from IDT (Coralville, IA, USA) and used to amplify the template DNA for the RNA probes using RT-PCR. Digoxigenin-labeled RNA probes were transcribed in vitro from the purified PCR product with T7 RNA polymerase (Roche Diagnostics, Laval, QC, Canada)^66^. Probe quality was confirmed by agarose gel electrophoresis. The probes were then stored at -80 °C.

#### Whole-mount patellar preparations

Preparations were fixed for 1 h in 4% Paraformaldehyde, dehydrated in a series of methanol solutions in 0.1% Triton X-100 in PBS (PBST) and stored at  − 20 °C. The tissue was rehydrated through methanol series in PBST followed by a protocol previously described in detail^66^. In the end, 1:8000 dilution of anti-Digoxigenin-Alkaline-Phosphatase, Fab-fragment (Roche) antibody reaction was visualized using NTB/BCIP^66^. The complete experimental protocols were repeated at least twice for each of the 12 antisense and sense (control) probes (Supplementary Table 1) using hybridization temperatures of 60 °C and 63 °C. In the end, the hypodermis bearing the sensory organs, was carefully detached from the cuticle, and mounted on a microscope slide in 70% glycerol. Previously confirmed probes were used as positive controls.

#### CNS preparations

The antisense and sense probes for *CsPiezo* (Supplementary Table 1) were tested on sections of the *C. salei* CNS. Paraformaldehyde fixed CNS was dehydrated in methanol series in PBST, stored at − 20 °C and then rehydrated in a series of methanol solutions in PBST. The CNS was then embedded into albumin-gelatin mixture, hardened with formaldehyde and glutaraldehyde as described earlier^67^. The blocks were sectioned by vibratome (Leica VT1000S, Leica Biosystems, Wetzlar, Germany), 30–40 µm thick horizontal sections were collected and processed as floating sections using the same protocol as for the whole-mount patellar preparations^66,67^. Before the final dye reaction, the sections were placed on microscope slides and allowed to dry at 37 °C. Warm glycerol/gelatin media was used to mount the coverslips on slides. Samples were photographed with an Axiovert microscope and Axioscope camera (Zeiss, Oberkochen, Germany) and the images were processed using Photoshop CC software (Adobe Systems, San Jose, CA, USA).

### Generation of a polyclonal antibody against the CsPiezo and Western blot analysis

Peptide sequence NIKLVDEQPRKVSGEVDDVE representing residues 1483–1502 of *C. salei* Piezo protein was synthesized and used to generate a polyclonal antibody in rabbits by Biomatik (Cambridge, ON, Canada). Affinity purified antibody was used in all experiments. The CsPiezo antibody was tested in Western blot on *C. salei* CNS. Freshly dissected CNS was homogenized in RIPA lysis buffer using BioMasher single use homogenizers with the Powermasher Pestle motor (Diagnocine LLC, Hackensack, NJ, USA). The homogenate was incubated on ice for 20 min followed by centrifugation at 16,000 g at 4 °C for 20 min. Standard SDS-PAGE and Western blotting methods were used^44^. Resulting membranes were probed with the CsPiezo antibody (1:1000) in blocking solutions (Tris buffered saline with 0.1% Tween-20 and 5% skimmed milk powder) followed by incubation in 1:20,000 dilution of a peroxidase conjugated goat-anti-rabbit secondary antibody. Immunoreactive protein bands were visualized using Clarity Western ECL kit (BioRad, Mississauga, ON, Canada). The log molecular weights of the protein standards were blotted to extrapolate the weight of the band detected by the antibody. For control, peptide competition was performed by preabsorbing the CsPiezo antibody for 1 h at room temperature in fivefold excess of the peptide (by weight) before the Western blot protocol.

### Immunocytochemistry

#### Patellar preparations

Similar whole mount preparations were prepared as for in situ hybridization. The tissue was permeabilized in 0.1% Saponin in PBS and processed using standard protocol^44^. Cell nuclei were stained with 1 µg/mL of 4′,6-diamidino-2-phenylindolein (DAPI) in PBS. In the end, the hypodermis containing the leg nerves, and several mechanosensilla, was detached from the cuticle and mounted on a microscope slide in Mowiol mounting medium^68^. The primary antibodies were the polyclonal rabbit CsPiezo (1:2000) and the monoclonal antibody against *Drosophila* synapsin (SYNORF 1, 1:100 dilution)^69^ that has been tested previously in *C. salei*^39,44,68^. Secondary antibodies were Cy-3 conjugated goat-anti-rabbit IgG for the CsPiezo, and AlexaFluor 488 conjugated goat-anti-mouse IgG for anti-synapsin, both at 1:500 concentrations. For control, the CsPiezo antibody was preabsorbed in fivefold excess of the blocking peptide for 1 h before the samples were incubated in this antibody solution.

#### CNS preparation

The CNS dissection and fixation were performed as described for in situ hybridization followed by embedding into albumin-gelatin mixture, except that glutaraldehyde was omitted to avoid autofluorescence, and formaldehyde was used for hardening the block. Horizontal 60 µm sections were cut using a Leica vibratome and processed as floating sections. Similar immunolabeling protocol as described for patellar preparations was used with the CsPiezo as primary antibody and CY-3 as the secondary antibody. DyLight 488 Phalloidin (Cell Signaling Technology) was used to stain actin filaments and DAPI to stain the cell nuclei. The sections were mounted in Mowiol mixture on a microscope slide.

Digital image acquisition was done using a LSM 710 Meta laser-scanning confocal microscope (Zeiss). Digital images of 1 µm optical sections were captured processed using Zen software (Zeiss) and the final images were prepared with Adobe Photoshop CC.

### Electrophysiology

We used two types of VS-3 preparations in electrophysiological experiments. In cuticular preparations, the hypodermis with the VS-3 neurons, is attached to the cuticle allowing mechanical stimulation of the slits as described previously in detail^31,33^. For hypodermis preparations, the patellar hypodermis with the VS-3 neurons was detached from the cuticle, mounted on a coverslip, and placed in a recording chamber^44,47^. Both preparations were superfused with spider saline. Neurons were observed using Axioscop 2FS Plus (Zeiss) microscopes. Sharp borosilicate glass microelectrodes were pulled with a P-2000 laser puller (Sutter Instrument, Novato, CA, USA) and filled with 3 M KCl. Recordings were made in discontinuous single electrode current- or voltage- clamp mode using SEC-10L amplifiers (npi electronic, Tamm, Germany)^44,47,68^. All experiments were controlled by an IBM-compatible computer using custom written software.

We used a custom-made borosilicate glass probe for mechanical stimulation^31,33^. The probe was mounted on a P-841.10 piezoelectric stimulator driven by an E-505.00 LVPZT amplifier (Physik Instrumente, Auburn, MA). The stimulator was mounted on a micromanipulator (SD instruments, San Diego, CA) to position the probe tip underneath the VS-3 slits.

For voltage-clamp experiments, the voltage-activated Na^+^ currents were blocked by 1 µM TTX. We tested two putative blockers of mechanotransduction channels; 10 µM Ruthenium Red and a synthetic D-amino acid form analog of the tarantula toxin GsMTx4 (5 µM), generously provided by Thomas Suchyna (University of Buffalo, NY). These blockers were diluted in spider saline with TTX and manually added to the preparation chamber via a syringe and plastic tubing completely replacing the bath solution. Fresh solution was added every 10–15 min during an hour-long experiment.

### Statistical analysis

Data from the electrophysiological experiments with Ruthenium Red and GsMTx4 was tested for normality using the d’Agostino-Pearson test. To test if the changes were statistically significant, t-test for paired samples was used.

## Supplementary Information

Supplementary Information

## Data Availability

All data generated or analysed during this study are included in this published article (and its Supplementary Information files).
