# Supplementary material for: Mechanotransduction channel Piezo is widely expressed in the spider, Cupiennius salei, mechanosensory neurons and central nervous system
Source: Sci Rep. 2021 Apr 12;11:7994. doi: 10.1038/s41598-021-87202-1 (PMC8042031; doi:10.1038/s41598-021-87202-1)
Supplement: Supplementary file 1 — Supplementary Information [file 41598_2021_87202_MOESM1_ESM.docx]

**Mechanotransduction channel Piezo is widely expressed in the spider, Cupiennius salei, mechanosensory neurons and central nervous systems**

Jessica A.G. Johnson, Hongxia Liu, Ulli Höger, Samantha M. Rogers, Kajanan Sivapalan, Andrew S. French and Päivi H. Torkkeli

**Supplementary Table 1:** Primer sequences used for generation of RNA probes for in-situ hybridization.

| **Name/ID** | **Accession No.** | **Antisense Primers** | **Sense Primers** |
| --- | --- | --- | --- |
| *CsENaC-A*  *CSH-477* | N/A | **F:**CATCTTCTGCCGTCTCCTTATC **R:**TAATACGACTCACTATAGGGGTCTTCGATAGCCATTCTTCCTAC | **F:**TAATACGACTCACTATAGGGCATCTTCTGCCGTCTCCTTATC **R:**GTCTTCGATAGCCATTCTTCCTAC |
| *CsENaC-B*  *CSH-449* | N/A | **F:**TTCACAGCTTGTAACATCCAGTG **R:**TAATACGACTCACTATAGGGAGCTCACCTGATTTCTTTCCG | **F:**TAATACGACTCACTATAGGGTTCACAGCTTGTAACATCCAGTG **R:**AGCTCACCTGATTTCTTTCCG |
| *CsENaC-C*  *CSH-285* | GAKT01000103 | **F:**CCAATGCTCAGCCTTATACGACTG **R:**TAATACGACTCACTATAGGGCTCCATTTCAAGCACCATGTG | **F:**TAATACGACTCACTATAGGCCAATGCTCAGCCTTATACGACTG **R:**GCTCCATTTCAAGCACCATGTG |
| *CsENaC-D*  *CSH-287* | GAKT01000105 | **F:**TACTACTGCGACGGAAACAGGTCC **R:**TAATACGACTCACTATAGGGAACGACAGCCCTCAAGTAGTTCCTC | **F:**TAATACGACTCACTATAGGGTACTACTGCGACGGAAACAGGTCC **R:**AACGACAGCCCTCAAGTAGTTCCTC |
| *CsNompC1*  *(TRPN*)  *CSH-283* | GAKT01000102 | **F:**GTATTGCGACGCACTGTATAGCC **R:**TAATACGACTCACTATAGGGCTTCTTGTGACTCGTCTCTGCCA | **F:**TAATACGACTCACTATAGGGGTATTGCGACGCACTGTATAGCC **R:**CTTCTTGTGACTCGTCTCTGCCA |
| *CsNompC2*  *(TRPN)*  *CSH-514* | N/A | **F:**ATCACCTGTCGGTCCTTAGA  **R:**TAATACGACTCACTATAGGGCCCTGAAAGGTGGATGAGTATG | **F:**TAATACGACTCACTATAGGGATCACCTGTCGGTCCTTAGA  **R:**CCCTGAAAGGTGGATGAGTATG |
| *CsIav*  *(TRPV)*  *CSH-523* | N/A | **F:**CATGTCGAGTGTTGGAGGATAG  **R:**TAATACGACTCACTATAGGGCAGGTATAACAGGTGCTGGATT | **F:**TAATACGACTCACTATAGGGCATGTCGAGTGTTGGAGGATAG  **R:**CAGGTATAACAGGTGCTGGATT |
| *CsNan*  *(TRPV)*  *CSH-526* | N/A | **F:**GACGGCACCACTGTAGAAATAA  **R:**TAATACGACTCACTATAGGGCCCAGGACAAACACCAGATAAA | **F:**TAATACGACTCACTATAGGGGACGGCACCACTGTAGAAATAA  **R:**CCCAGGACAAACACCAGATAAA |
| *CsTRP short*  *CSH-478* | N/A | **F:**TGATGTCGATGAAGACCTTCAGCAG **R:**TAATACGACTCACTATAGGGCTAACTGCAATACAGCAGCTCTCTCG | **F:**TAATACGACTCACTATAGGGTGATGTCGATGAAGACCTTCAGCAG **R:**CTAACTGCAATACAGCAGCTCTCTCG |
| *CsTMC5*  *CSH-369* | GBFC01000028 | **F:**GATCCTGGTCTGTGAATGAGGC **R:**TAATACGACTCACTATAGGGCCAGGCTGATGAGAAGGTATCAG | **F:**TAATACGACTCACTATAGGGGATCCTGGTCTGTGAATGAGGC **R:**CCAGGCTGATGAGAAGGTATCAG |
| *CsTMC7*  *CSH-368* | GBFC01000027 | **F:**ACTGTAATAGAAGCCGGTCATGC **R:**TAATACGACTCACTATAGGGCTCTTGCTTGAATAGGGCACATC | **F:**TAATACGACTCACTATAGGGACTGTAATAGAAGCCGGTCATGC **R:**CTCTTGCTTGAATAGGGCACATC |
| *CsPiezo*  *CSH-288* | GAKT01000106 | **F:**GCAATGGAGTAGACTTCAAACTGCTG **R:**TAATACGACTCACTATAGGGAGCACTCTATCCACGTATGGCATATC | **F:**TAATACGACTCACTATAGGGGCAATGGAGTAGACTTCAAACTGCTG **R:**AGCACTCTATCCACGTATGGCATATC |

Accession No. = GenBank accession number; ID = Laboratory identification # (http://asf-pht.medicine.dal.ca/CSH-Web/); TMC = Transmembrane Channel-Like; ENaC = Epithelial Sodium Channel; TRP = Transient Receptor Potential; Iav = Inacxtive; Nan = Nanchung; F = forward polarity, R = reverse polarity

.
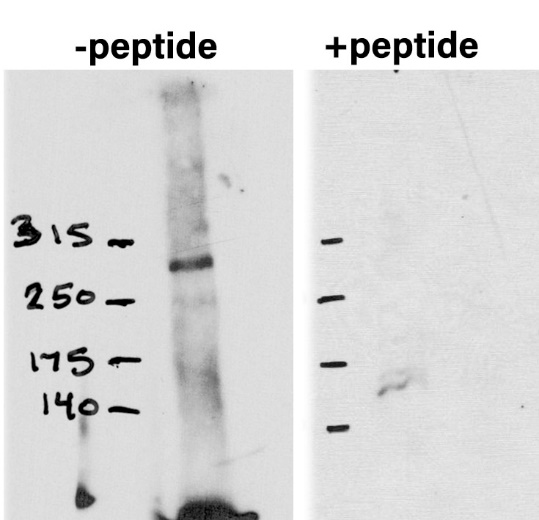


**Supplementary Figure S1**. Full-length unprocessed pictures of the Western blots in Figure 2e.
